# Locality-Based Cache Management and Warp Scheduling for Reducing Cache Contention in GPU

**DOI:** 10.3390/mi12101262

**Published:** 2021-10-17

**Authors:** Juan Fang, Zelin Wei, Huijing Yang

**Affiliations:** Faculty of Information Technology, Beijing University of Technology, Beijing 100124, China; weizelin@emails.bjut.edu.cn (Z.W.); yangkx@emails.bjut.edu.cn (H.Y.)

**Keywords:** GPGPU, cache management, warp scheduling

## Abstract

GPGPUs has gradually become a mainstream acceleration component in high-performance computing. The long latency of memory operations is the bottleneck of GPU performance. In the GPU, multiple threads are divided into one warp for scheduling and execution. The L1 data caches have little capacity, while multiple warps share one small cache. That makes the cache suffer a large amount of cache contention and pipeline stall. We propose Locality-Based Cache Management (LCM), combined with the Locality-Based Warp Scheduling (LWS), to reduce cache contention and improve GPU performance. Each load instruction can be divided into three types according to locality: only used once as streaming data locality, accessed multiple times in the same warp as intra-warp locality, and accessed in different warps as inter-warp data locality. According to the locality of the load instruction, LWS applies cache bypass to the streaming locality request to improve the cache utilization rate, extend inter-warp memory request coalescing to make full use of the inter-warp locality, and combine with the LWS to alleviate cache contention. LCM and LWS can effectively improve cache performance, thereby improving overall GPU performance. Through experimental evaluation, our LCM and LWS can obtain an average performance improvement of 26% over baseline GPU.

## 1. Introduction

The general-purpose graphics processing unit (GPGPU) is one of the most mainstream acceleration components in the field of throughput-oriented high-performance computing [1,2]. The high performance of GPGPU lies in its huge multi-threaded architecture. By quickly switching the context between different threads, it can hide the long delay caused by operations such as memory access. In addition, compared with the CPU, GPGPU has more processing units to support its single instruction multi-thread execution model. That is, all threads execute the same instruction on different data [3], which can provide better performance improvements and multi-core CPU efficiency. In order to make a large number of threads run efficiently, multiple consecutive threads are divided into a thread group, called warp or wavefront [4]. Warp is the basic unit for task scheduling and execution. CUDA [5] and OpenCL [6] make GPU-based general-purpose computing widely used in many disciplines, such as image processing, pattern recognition, and neural networks.

The special multi-thread execution mode of GPUs determines that the memory access efficiency plays a crucial role in the performance of the application. Since a large number of threads may simultaneously issue memory access requests at the same time, if the design of the storage hierarchy cannot effectively respond to this access pattern, causing these memory access requests to flow to the off-chip DRAM, it will cause a large number of threads to wait for data, thereby significantly reducing GPU computing effectiveness. If the application’s memory access behavior cannot reasonably match the design of the on-chip GPU storage hierarchy, and the potential data locality is not well used, GPU performance will be limited. Therefore, GPU memory analysis and optimization have become issues of high concern in GPU performance optimization.

The main purpose of this paper is to improve GPU performance by reducing cache contention. Cache contention refers to the case when two processing units try to update the same cache lines. In the GPU data cache, the L1 data cache is especially significant for reducing memory access latency and meet high bandwidth requirements. Due to the large number of multi-threaded tasks in the GPU, the cache space required by the application often exceeds the L1D capacity. For example, each streaming multiprocessor (SM) in the Fermi and Kepler architecture supports 1536 threads and 2048 threads, respectively. However, the capacity of the L1 data cache shared by these threads is only 16 KB or 48 KB [7].

In order to reduce memory access, the GPU integrates the memory coalescing unit into the load/store unit (LDST unit), so that once instructions needing to access memory are executed, many memory requests generated by a single warp would be coalesced into a few memory transactions, improving the efficiency of memory access. However, it is only when the memory requests from a single warp are regular, coalesced into several cache blocks, that the memory coalescing effectively improves performance. Current GPGPU applications always have irregular memory requests. The requests from the same warp cannot always be coalesced. This non-coalesced memory access often leads to memory divergence [8,9]. That is, in instruction related to memory operation, some threads in the same warp return fast due to cache hits, while the other threads need to wait a long time because of being missed in cache. All threads in the warp must wait for the slowest memory access to complete because of the Single Instruction Multiple Data (SIMD) execution model of GPU. SIMD units refer to hardware components that perform the same operation on multiple data operands concurrently. The instructions that have coalesced will also cause a lot of memory access, which will aggravate L1D contention, affecting the performance [10,11]. This means the L1 data cache often becomes a performance bottleneck.

How to improve cache utilization and improve cache performance is the key to improving the GPU processing power, improving the resource utilization rate, and optimizing the system operating efficiency. This paper proposes a GPU cache management strategy based on data locality features, combined with the warp scheduling strategy to improve GPU performance. Each load instruction can be divided into three categories. According to the locality characteristics of the load instruction, apply cache bypass to the streaming locality request to improve the cache utilization rate; at the same time, simultaneously make full use of the data locality between the warps through the inter-warp memory request coalescing; combined with the locality-based warp scheduling to further alleviate cache contention. By adopting different optimization strategies for different local characteristics, the GPU cache management strategy based on data locality types can effectively improve cache performance and alleviate pipeline congestion, thereby improving overall GPU performance. The main contributions of our work are the following:

- This paper introduces the challenges encountered by massive memory accesses under the massive multi-threaded architecture of GPGPU, and data cache plays a vital role in it. We analyzed the cache contention in GPGPU applications, and we can take advantage of the different locality characteristics.
- We propose Locality-Based Cache Management (LCM), combined with the Locality-Based Warp Scheduling (LWS), to improve GPU performance. First, according to the locality characteristics of the load instruction, improve the cache utilization by bypassing the streaming data, and extended inter-warp memory request coalescing to make full use of inter-warp data locality. LWS alleviates cache contention by applying different warp scheduling strategies according to cache performance. By adopting different optimization strategies for different locality characteristics, LCM and LWS can effectively improve cache utilization.
- In the GPGPU system built by the GPGPU-Sim simulator, we evaluate the LCM and LWS. The experimental results show that the LCM and LWS can improve system performance.

The rest of this paper is organized as follows: Section 2 introduces the baseline GPU architecture and memory coalescing and then analyzes GPU memory access inefficiency. Section 3 describes the proposed Locality-Based Cache Management. Section 4 introduces the proposed Locality-Based Warp Scheduling. Section 5 analyzes the experimental results. Section 6 discusses the related work of cache management and warp scheduling. Finally, Section 7 concludes the paper.

## 2. Related Work

Cache management and replacement schemes designed for CPUs are often not suitable for GPUs [12,13] because they are not designed for large-scale multithreading of GPUs. Among various GPU cache management strategies, cache bypass is an effective technique to alleviate cache contention. Methods have been proposed for CPU [14,15]. The CPU’s cache bypass strategy mainly uses the cache hit rate as a guide criterion for cache bypass, which is usually used for the last level of cache. However, on the GPU [15,16,17,18,19,20,21,22], the model based on the cache hit rate does not always perform well due to its unique architectural characteristics, including a lot of parallelisms, resource congestion, and memory divergence. A model-driven approach was developed by [23] which dynamically estimates the impact of cache contention and resource congestion as a function of the number of warps/thread blocks (TBs) to bypass the cache. Xie et al. [17] proposed a compiler-based method to access or bypass the cache by analyzing reuse distance and memory traffic. [22] proposed a locality-driven dynamic cache bypassing technique, which exploits the locality information to adjust the cache behavior at run-time.

Memory request coalescing technology can provide more efficient memory access [24,25] and improves GPU security [26]. For the GPU, the memory merge unit in the LDST unit is designed to coalesce requests to access consecutive 128 B data in the memory. Described in [24] is a compiler-based technique that better distributes the memory requests in time by re-organizing the static instruction stream. Pei et al. [25] proposed an equidistant memory access merging strategy, which merges memory access requests with long but equal memory access distances to reduce memory access requests and NoC transmitted data. The proposed memory access merging strategy can effectively reduce the number of memory requests and save data transmission overhead, but it is not optimized for memory access between warps and cannot make full use of the data locality between warps.

A lot of research on warp scheduler has been proposed. Rogers et al. [27] proposed a divergence-aware warp scheduling strategy (DAWS), which introduced a predictor to estimate the required capacity of L1 data cache to capture the warp locality in the loop based on online information in the warp. Sethia et al. [28] proposed MASCAR, which uses greedy scheduling techniques to detect memory saturation and limit the warp for sending memory requests at a short period of time. In order to improve the latency hiding ability, Do et al. [29] proposed a long-latency operation-based warp scheduler to improve GPU performance. Liang et al. [30] proposed coordinated static and dynamic cache bypassing. They also developed a bypass-aware warp scheduler to adaptively adjust the scheduling policy based on the cache performance. Li et al. [31] proposed priority-based cache allocation (PCAL) that provides preferential cache capacity to a subset of high-priority threads while simultaneously allowing lower priority threads to execute without contending for the cache, which is a codesign between the thread scheduler and cache allocation scheme to avoid cache contention without underutilizing other resources. However, the scheduling algorithm will destroy the locality to a certain extent and generate more off-chip memory access.

The efficiency of the storage hierarchy is a key performance factor. Existing research uses multiple methods to manage GPU cache to solve the problem of the GPU memory subsystem and cache management efficiency. In addition to the above methods, there are warp throttling [32] and memory scheduling strategy [33].

## 3. Background and Motivation

### 3.1. Baseline GPU Architecture

**Figure 1:**
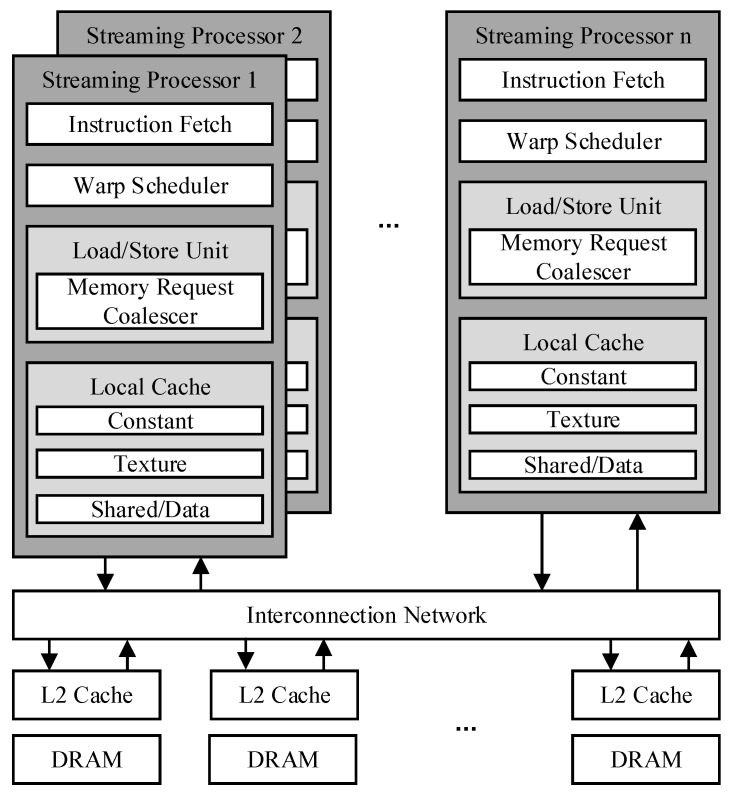
Baseline GPU architecture.

The baseline GPU architecture is composed of multiple SMs. The parallelism of GPUs is achieved by multiple SMs. Figure 1 shows a diagram of typical GPGPU architecture.

GPUs execute threads with the SIMT execution model. Every 32 threads are gathered into a warp. In general, each warp is a thread cluster composed of 32 threads and is the smallest scheduling unit of GPGPU. In the absence of branches, threads in a warp access different data and execute the same instructions. Performance can be significantly improved by making threads from the same warp execute the same code path and access neighboring addresses. The LDST unit is a functional unit responsible for load, store, and memory barrier instructions. It simultaneously processes 32 threads of warp as a functional unit.

The GPU memory hierarchy comprises register memory, L1 cache, shared L2 cache, and off-chip dynamic random-access memory (DRAM). There is an L1 cache in each SM. The L1 cache is private and accessed by the warps within the core. In contrast, the L2 cache can be shared by all cores. Warp running on any SM can access the L2 cache. The core interacts through the interconnection network. When the core sends a memory request to L2 cache, the intermediate network sends it to different memory ports on the L2 cache according to the address of the memory request and then accesses different cache blocks’ process data access. A GPU application will initiate one or more kernel functions to the GPU, and each kernel function will allocate one or more thread blocks co-operative thread arrays (CTA) to each SM for execution.

### 3.2. Baseline Memory Coalescing

**Figure 2:**
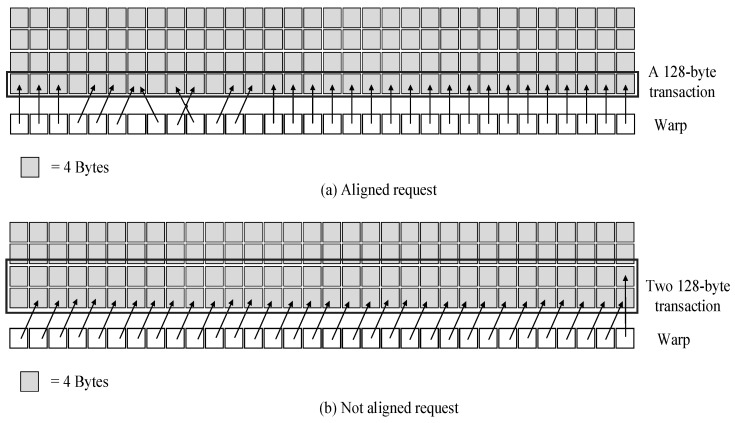
An example of baseline memory request coalescing.

Global memory resides in the device memory and can be accessed through 32 B, 64 B, or 128 B memory transactions. The memory transactions can only read or write 32 B, 64 B, or 128 B device memory segments aligned to their size. Examples of aligned and unaligned memory transactions are shown in Figure 2. The memory segment in Figure 2a is naturally aligned, and a 128-byte transaction can be sent, while the memory segment in Figure 2b is not, so that two 128-byte transactions will be sent.

When a warp accesses global memory, it coalesces the memory request of threads in the warp into fewer memory transactions. The coalescing is based on the size of the word, and the allocation of memory addresses in memory. For instance, the threads in a single warp access a continuous 4-byte data, a 128-byte data request will be sent to DRAM instead of 32 4-byte requests, which reduces the number of transactions between SIMT core and DRAM, and reduces the workload of the interconnection network, memory partitions, and DRAM.

Miss Information/Status Holding Registers (MSHR) store the address, size, type, and other information about requests. Once the memory controller returns the data needed by miss access, the information is used for re-execution. MSHR is also used to coalesce multiple requests for the same line to prevent the same request from being sent multiple times.

### 3.3. Memory Access Inefficiency

In the current GPU architecture, the cache capacity of the L1 data cache is not high. Nevertheless, the SIMD architecture will generate a lot of memory requests in a short period of time. Required memory capacity will even be 1 to 2 orders of magnitude larger than the L1 data cache. This leads to severe cache contention. One of the reasons for cache contention is the failure to make effective use of data locality. If you cannot distinguish the reuse of the data, the data may be ejected from the cache before hit in the cache when the application reuse distance is long. Data that will not be reused may occupy the cache, causing cache pollution. The second reason is conflict in the cache. Thirty-two threads executing concurrently in a warp may access the same cache set. The third reason is that stall will be caused by the lack of other resources, such as MSHR resources.

**Figure 3:**
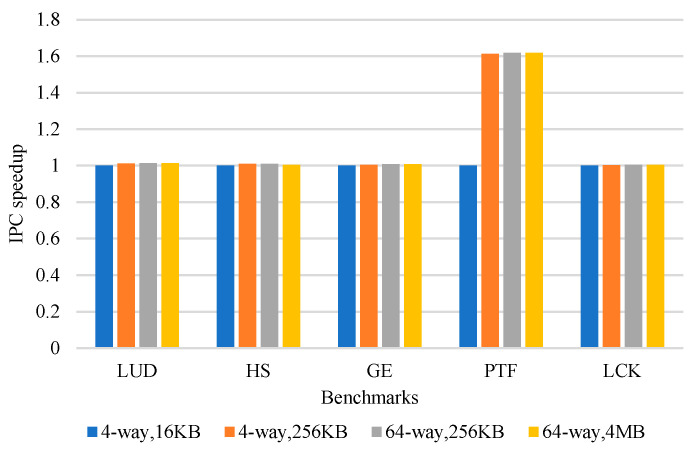
Normalized IPC speedup of different cache capacity and associativity.

In order to reduce cache contention, we experimentally observed the impact of increasing the capacity and associativity of L1 data cache on performance. The experimental results are shown in Figure 3. It can be seen from the experimental results that for some applications, increasing the cache capacity and associativity cannot effectively improve performance. Meanwhile, this will significantly increase the hardware cost, access latency, and power consumption. Therefore, we consider reducing the contention of the cache and improving the performance by making full use of data locality.

## 4. Locality-Based Cache Management

**Figure 4:**
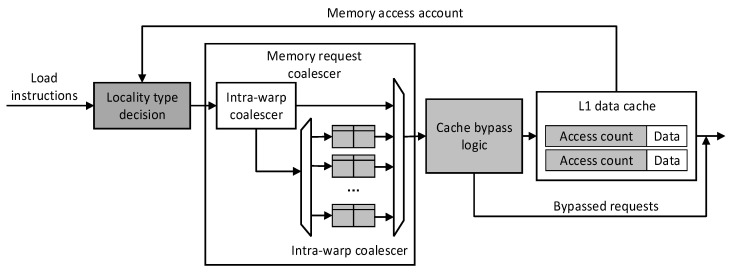
Overall diagram of Locality-Based Cache Management architecture.

The overall architecture diagram of Locality-Based Cache Management is shown in Figure 4. The gray components are the parts that are modified in this topic. Each load instruction classifies data locality by reading the access count in the cache, adding a merge queue between warps in the memory access merge unit, and making full use of the local merge memory access between warps. The cache bypass component makes data that are no longer reused bypass the cache, saving cache space. The warp scheduling strategy will be discussed in Section 4.

### 4.1. Criteria of Locality Type Decision

Classified by each global load instruction, there are three types of locality that can be exhibited by the data of load instruction: streaming locality, inter-warp locality, and intra-warp locality. The data may show both intra-warp locality and inter-warp locality at the same time.

Our classification method is like [34]. Streaming data refers to being used only once by a single warp and is no longer reused after being inserted into the cache, resulting in a waste of cache resources. Inter-warp locality referred to the data used by one warp and accessed again by one or multiple other warps. If the data are used multiple times by a single warp, the data locality is called inter-warp. A single load instruction can show both inter- and intra-warp locality at the same time.

Before applying specific strategies to different types of data locality, first, decide the load instructions’ locality types. The method to detect the locality type is based on the access count of the same warp and the access count of requests from other warps. The criteria of locality types are shown in Table 1. For example, if warp B accesses a cache line accessed by warp A earlier, the total access count is 2, and the intra-warp access count of warp A is 1. That is in line with the standard of inter-warp locality type. Then we can infer that the cache line shows the locality of inter-warp.

Every load instruction in a GPU application will tend to have a similar cache access mode because threads of a single core execute the same code [34]. Therefore, we can detect a warp in the global load instruction to classify the data locality of the load instruction. The type of locality detected by the monitored warp is considered the same as the other warps.

**Figure 5:**
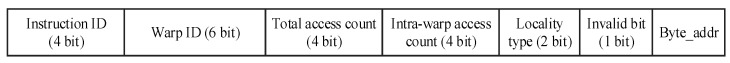
Entries of locality type detection formation table.

The table records the instruction ID as each global load instruction begins to execute. The instruction PC will be hashed to save the storage. When executing the instruction, the total access count of the data and the access count of the detected warp are recorded and updated synchronously. If the access request comes from the detected warp, the total access count and the access count of the same warp are both set as one. If the request comes from another warp, only the total access count is set as 1. When the same load instruction generates an address conflict or the total number of accesses exceeds the threshold, the current locality type is calculated, and the field of the invalid bit is set as 1. At that time, the locality-specific strategy can be adopted. Then release the entry and wait for the update. The locality type field consists of 2 bits, the first is set as 1 for the intra-warp locality, and the other is set as 1 for the inter-warp locality. If it is detected as streaming data, the locality type field is set as 00. In the L1 data cache, a field is needed to record the total access count to initialize the total access count of the information table entry when the detected warp accesses the data reaccessed by another warp. To save hardware overhead, we use the hash value of the PC as the instruction ID. Entries of the locality type detection formation table are shown in Figure 5.

### 4.2. Inter-Warp Memory Request Coalescing

Inter-warp memory request coalescing has been considered effective in previous work [35]. Our proposed inter-warp memory request coalescing is based on the detection results of the locality type, and only coalesce requests that access to data exhibit inter-warp locality, reducing the overhead of finding more opportunities to coalesce.

The baseline coalescing can only coalesce requests between threads of a single warp. To find more coalesce opportunities and improve data utilization, the inter-warp memory request coalesce is added, aiming to lookup requests issued by different warps and increase the coalesce window of the requests of threads in different warps.

The inter-warp coalesce queue constitutes an inter-warp coalescing window, coalescing requests from different warps. Requests from the intra-warp coalesce queue are mapped to one of the multiple queues based on a subset of their addresses. When the inter-warp coalescing queue receives a request, its cache line will match the cache line already in the queue, coalescing requests from the same cache line.

Several tags in the inter-warp coalesce queue are used to record the request information of a cache line. The warp ID, load instruction, and the mapping information in the cache line were saved in each tag. Once the queue received a request, a lookup is performed on the tag. Then the request will be inserted under the tag if matching. If there is no matching entry, a new entry is assigned when idle. In this work, only two tags per queue are reserved to reduce the number of tag lookups required and the times the memory request coalescer attempts to insert into the same queue, reducing overhead.

The inter-warp coalesce queue takes temporal information as the priority and evicts the requests that enter the queue first to ensure that requests will not stay in the queue for too long and will not destroy the temporal locality of the warp.

### 4.3. Locality-Based Bypassing

Many resources are occupied when fetching streaming data into the cache, such as cache lines and MSHR entries. However, streaming data will not be reused, so that the above resources will be wasted. Furthermore, some other data with a good locality in the application may be ejected from the cache. Cache bypass policy is a standard method to deal with such problems. Therefore, the method of processing the detected streaming data is the cache bypass policy. The locality-based bypassing directly provides data to the computing core from the L2 cache and its related interconnection network to improve resource utilization and reduce cache contention.

When the cache bypass component receives the request and reads the locality type as streaming, it directly sends the request to the L2 cache. Otherwise, the request will be sent to the L1 cache.

## 5. Locality-Based Warp Scheduling

### 5.1. Overview

The GTO scheduling strategy is now a commonly used warp scheduling strategy. It prioritizes all warps on the GPU according to time. The oldest warp always has the highest priority. This method can effectively retain data locality [8]. However, cache contention and pipeline stall both have a huge impact on GPU performance. To obtain the best performance, we introduced the locality type detection method mentioned in Section 3 and use different warp scheduling strategies for different locality types. Both consider locality and ease cache contention.

As mentioned in Section 3.3, the cache bypass policy bypasses the L1 cache based on the locality type. If the warp scheduler schedules many cached warps in a short time, it may cause cache contention. On the other hand, if the warp scheduler schedules many warps decided to bypass the cache when the cache is idle, it cannot fully utilize the data locality. We can alleviate cache contention by modifying the priority of streaming data and requests of other locality types at runtime based on performance. Therefore, we proposed a locality-based warp scheduling policy. When the cache is busy, the bypassed warp is preferentially scheduled to alleviate the pressure; when the cache is idle, the cached warp is preferentially scheduled; otherwise, the default GTO scheduling policy is used.

### 5.2. Implementation

When the warp scheduler needs to decide the warp to be issued, first check its locality type. If it is streaming data, it means it will be bypassed the cache. Then check if the cache is busy or idle. In order to quantify the performance of the cache, we propose a Cache Utilization Score (CUS) as a basis for judgment. As shown in Equation (1), the numerator of CUS is the product of the number of cache hits and the L2 cache latency and represents the speedup brought by cache hits. The denominator of CUS is the product of the number of cache misses and the pipeline stall and represents the degree of cache contention.

(1) $$\mathrm{CUS}=\frac{n_{\mathrm{hit}}\cdot {\mathrm{Latency}}_{L2}}{n_{\mathrm{miss}}\cdot {\mathrm{Stall}}_{\mathrm{net}}}$$

To keep a certain balance between cache contention and full use of the locality, when the CUS is too high or too low, warp scheduling strategies need to be changed. When the CUS value is greater than the threshold H, we consider that the cache is idle and has good performance, and the cached warp can be scheduled faster to make full use of the cache. When the CUS value is less than the threshold L, we think that the cache is contention and busy; scheduling cached warp will increase the contention. Therefore, the bypassed warp should be scheduled first to ease the pressure of the cache. If the CUS value is between the threshold H and the threshold L, we believe that priority scheduling of cached warp or bypassed warp will not make a big difference. Therefore, the default GTO warp scheduling strategy is adopted.

The warp scheduler first checks whether the load instruction executed by the warp is marked as streaming. Divide streaming warp into a group, and other warp into a group. Then compare CUS with both thresholds. Similarly, if the CUS value is less than the threshold H, the streaming warp group is set to a high priority group. If the CUS value is greater than the threshold L, the other warp groups are set as high priority groups. Use GTO scheduling strategies within the group. Otherwise, the GTO scheduling strategy is used within the scope of the two groups.

We determined the values of threshold H and threshold L for better performance through experiments. In the experimental setup of this paper, we set the threshold H and threshold L to 1 and 0.2. Compared with the default GTO scheduling strategy, our LWS can make full use of local type information at runtime, alleviate cache contention, and make full use of the cache.

## 6. Evaluation

### 6.1. Methodology

We implemented our strategy in GPGPU-Sim version 3.2.3 [30] and evaluated it. GPGPU-sim is a cycle-level GPGPU simulator, which is widely used in the research of GPU architecture. The configuration we used is shown in Table 2 and is based on the GPU of NVIDIA Kepler architecture, like GTX 780.

The details of the benchmark we used are shown in Table 3, which is selected from PolyBench [36] and Rodinia [37] benchmark suite.

### 6.2. Results

#### 6.2.1. Speedup

**Figure 6:**
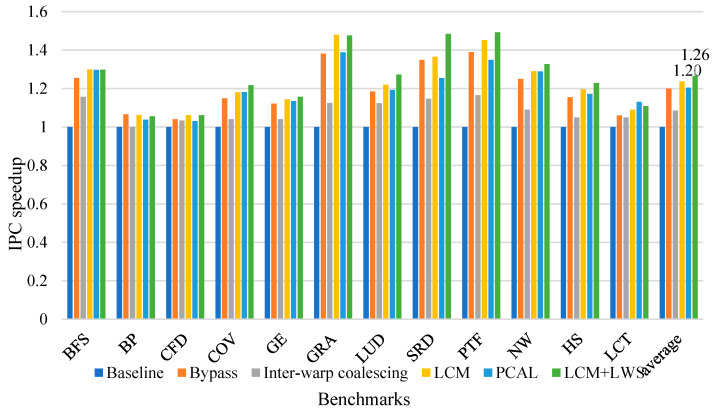
Speedup of LCM, LWS over baseline GPU.

Figure 6 shows our LCM and LWS IPC speedup over baseline GPU. We use the instruction per cycle (IPC) executed as an evaluation indicator. We also tested the speedup of combining separate cache bypass and inter-warp memory requests. PCAL shows the performance of the PCAL mechanism proposed in [31]. We can infer from the Figure 6 that our LCM performance achieves an improvement of 23% on average. A separate cache bypass strategy improves GPU performance by 19% on average, while inter-warp memory request merge provides 9% speedup. Combined with LWS, our strategy can provide an average speedup of 26%. Compared to PCAL, LCM and LWS provide 6% better performance. PCAL reduces the amount of cache thrashing, while LCM and LWS shows more speedup by reducing cache contention.

By bypassing the streaming data, the cache bypass policy alleviates cache contention while protecting other cache lines, providing more opportunities for data with a better locality. Because streaming data is almost no longer reused or has a longer reuse distance, the cache cannot be used effectively. The cache bypass policy has the best results for applications with high cache sensitivity, such as GRA and PTF, which provide up to 38% performance improvement. Inter-warp memory request coalescer provides additional memory consolidation opportunities for data with inter-warp locality over baseline coalescer. As a result, performance has improved by an average of 9%. The LCM, which combines the cache bypass strategy and inter-warp memory access merger, has shown better performance than both separate policies.

Baseline GPU uses the default GTO warp scheduling strategy. LWS considers streaming data that bypassed cache, which can further alleviate cache contention over the cache bypass strategy. LCM combined with LWS, has achieved a total performance improvement of 26%, and almost all applications can benefit from LWS. The experimental results prove that our strategy is effective and can bring significant performance improvements.

#### 6.2.2. L1 Cache Miss Rate

**Figure 7:**
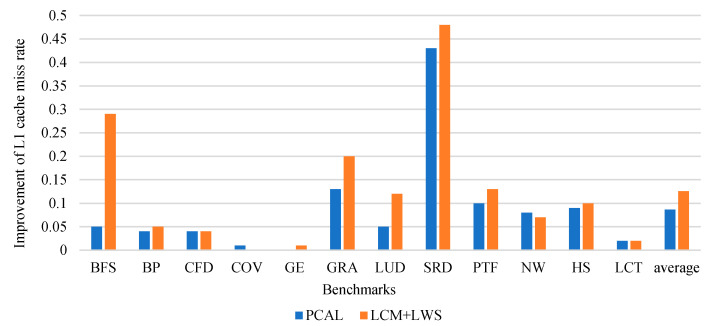
Reduction in the miss rate of LCM for the L1 data cache.

From Figure 7, we can see the reduction in the miss rate of LCM for the L1 data cache compare to PCAL. From the result, we can see that the miss rate has decreased by an average of 13%, and 4% better than PCAL. The cache bypass strategy effectively bypasses L1 cache for requests with poor locality, enables cache lines to be reused more efficiently, and protects data with high locality. Our strategy is mainly to reduce the cache miss rate and reduce cache stall to improve performance. Memory access-intensive caches such as COV mainly reduce cache stall to improve performance, so the optimization effect on cache miss rate is not obvious.

#### 6.2.3. Sensitivity to Cache Size

**Figure 8:**
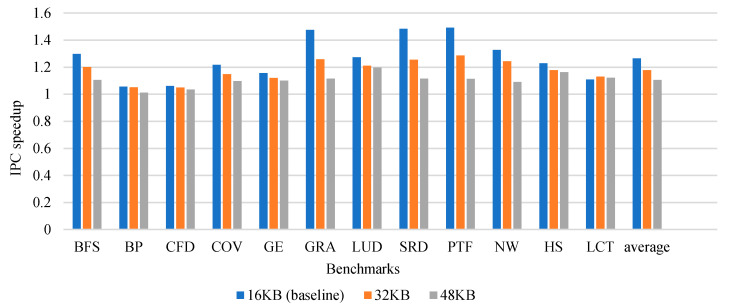
IPC speedup over different cache capacity.

Figure 8 shows the performance speedup over different sizes of cache. For 16-KB, 32-KB, and 48-KB caches, it achieves average speedups of 26%, 18%, and 11%, respectively. From the experimental results, we can infer from the result that the larger the cache size, the smaller the performance improvement. This is because an increase in the size of the cache will alleviate cache contention, and it is less likely to reuse data that are not very long away from the cache. However, for most applications, our LCM and LWS can improve performance.

### 6.3. Hardware Overhead

Table 4 shows the hardware overhead required to implement LCM and LWS. In addition to the tags mentioned in Chapter 2 and the third sheet, you also need to store information for unfinished requests, so the MSHR metadata table is added. The total hardware overhead is about 1.6 KB.

## 7. Conclusions

GPGPU has become the mainstream trend currently, and the memory system occupies a very important position in it. The optimization of the cache management strategy also plays an important role in the optimization of the overall performance. How to improve the cache utilization and improve the cache performance is the key to improving the GPU processing power, improving the resource utilization, and optimizing the system operating efficiency. In order to reduce cache contention, we proposed Locality-Based Cache Management and Locality-Based Warp Scheduling. LCM and LWS consider the use of different types of locality and further optimizes the warp scheduling strategy. Each load instruction classifies data locality by reading the access count in the cache, adding several inter-warp coalesce queues, and making full use of the inter-warp locality. The cache bypass policy makes data that are no longer reused bypass the cache to save cache resources. LWS selectively schedules warps accessing streaming data or other warp according to the cache and the busyness of the Internet. The experiment was implemented in an extended GPGPU-Sim simulator. Experimental results show that the LCM and LWS we proposed can effectively improve system performance, compared with the benchmark strategy, reduce the L1 cache miss rate, and obtain an average performance improvement of 26%.

In the GPU, fast thread switching increases the difficulty of prefetching data. If the data cannot be prefetched, it is likely to hurt performance. The next step can be to use the most suitable prefetch strategy for different memory access characteristics, and improve the accuracy and coverage of prefetching.

## Figures and Tables

**Table 1:** Criteria of locality type.

Type	Intra-Warp Access Count	Intra-Warp Access Count
Streaming	1	1
Inter-warp	1 ≤ Intra-warp access count < Total access count	>1
Intra-warp	1 < Intra-warp access count ≤ Intra-warp access count	>1

**Table 2:** GPGPU-sim baseline configuration.

Parameter	Configuration
CUDA cores and SMs	192 per SM, 16 SMs, 876 MHz
SIMT width	32
Warps and CTAs	64 warps/SM, 16 CTAs/SM
Scheduler	GTO scheduler, 2 per SM
Register file	256 KB
L1 data cache	16 KB, 4-way associative, 128 B/line, 4-way, LRU,64 MSHRs
L2 cache	1536 KB total, 16-way associative
DRAM	GDDR5 1674 MHz, 6 channels, 8 banks per rank, burst length 8

**Table 3:** Information of benchmarks.

Abbr.	Description	Benchmark Suite
BFS	Breadth-First Search	Rodinia [29]
BP	Backprop	Rodinia [29]
CFD	CFD Solver	Rodinia [29]
COV	Convolution	PolyBench [28]
GE	Gaussian Elimination	PolyBench [28]
GRA	GRA	PolyBench [28]
LUD	LU Decomposition	Rodinia [29]
SRD	Speckle Reducing Anisotropic Diffusion	Rodinia [29]
PTF	Particular Filter	Rodinia [29]
NW	Needleman-Wunsch	Rodinia [29]
HS	Hot Spot	Rodinia [29]
LCT	Leukocyte	Rodinia [29]

**Table 4:** Hardware overhead.

Extended in L1D	6 bits per tag, 128 lines
Locality information	21 bits per tag, 32 lines
Load alias table	32 bits per entry, 16 entries
Inter-warp coalescing queues	124 bits per tag, 64 lines
MSHR metadata table	23 bits per entry, 128 entries
CUS information	16 bits
Total	1606 Bytes

## References

[1] Devi J., Kumar J. (2019). The Computational Efficiency of Monte Carlo Breakage of Articles using Serial and Parallel Processing: A Comparison. Int. J. Adv. Comput. Sci. Appl..

[2] Cheng L., Li T. Efficient data redistribution to speedup big data analytics in large systems. Proceedings of the IEEE International Conference on High Performance Computing.

[3] Lindholm E., Nickolls J., Oberman S., Montrym J. (2008). NVIDIA Tesla: A Unified Graphics and Computing Architecture. IEEE Micro.

[4] He Y., Zhang Y., Shen F. (2016). Thread scheduling optimization of general purpose graphics processing unit: A survey. J. Comput..

[5] Cheng J., McKercher T. (2014). Professional CUDA C Programming.

[6] Du P., Weber R., Luszczek P., Tomov S., Peterson G., Dongarra J. (2012). From CUDA to OpenCL: Towards a performance-portable solution for multi-platform GPU programming. Parallel Comput..

[7] Zhao C., Wang F., Lin Z., Zhou H., Zheng N. Selectively GPU cache bypassing for un-coalesced loads. Proceedings of the 2016 IEEE 22nd International Conference on Parallel and Distributed Systems (ICPADS).

[8] Burtscher M., Nasre R., Pingali K. A quantitative study of irregular programs on GPUs. Proceedings of the 2012 IEEE International Symposium on Workload Characterization (IISWC).

[9] O’Neil M.A., Burtscher M. Microarchitectural performance characterization of irregular GPU kernels. Proceedings of the 2014 IEEE International Symposium on Workload Characterization (IISWC).

[10] Fauzia N., Pouchet L.-N., Sadayappan P. Characterizing and enhancing global memory data coalescing on GPUs. Proceedings of the 2015 IEEE/ACM International Symposium on Code Generation and Optimization (CGO).

[11] Wu B., Zhao Z., Zhang E.Z., Jiang Y., Shen X. Complexity analysis and algorithm design for reorganizing data to minimize non-coalesced memory accesses on GPU. Proceedings of the 18th ACM SIGPLAN Symposium on Principles and Practice of Parallel Programming.

[12] Jain A., Lin C. Back to the future: Leveraging Belady’s algorithm for improved cache replacement. Proceedings of the 2016 ACM/IEEE 43rd Annual International Symposium on Computer Architecture (ISCA).

[13] Teran E., Wang Z., Jiménez D.A. Perceptron learning for reuse prediction. Proceedings of the 49th Annual IEEE/ACM International Symposium on Microarchitecture (MICRO).

[14] Lee B., Kim K., Chung E.Y. (2017). Replacement policy adaptable miss curve estimation for efficient cache partitioning. IEEE Trans. Comput.-Aided Des. Integr. Circuits Syst..

[15] Jia W., Shaw K.A., Martonosi M. Characterizing and improving the use of demand-fetched caches in GPUs. Proceedings of the ACM International Conference on Supercomputing (ICS’12).

[16] Dublish S., Nagarajan V., Topham N. (2016). Cooperative Caching for GPUs. ACM Trans. Arch. Code Optim..

[17] Liang Y., Xie X., Sun G., Chen D. (2015). An Efficient Compiler Framework for Cache Bypassing on GPUs. IEEE Trans. Comput. Des. Integr. Circuits Syst..

[18] Jia W., Shaw K.A., Martonosi M. MRPB: Memory request prioritization for massively parallel processors. Proceedings of the 2014 IEEE 20th International Symposium on High Performance Computer Architecture (HPCA).

[19] Fang J., Zhang X., Liu S., Chang Z. (2019). Miss-aware LLC buffer management strategy based on heterogeneous multi-core. J. Supercomput..

[20] Zheng Z., Wang Z., Lipasti M. (2014). Adaptive Cache and Concurrency Allocation on GPGPUs. IEEE Comput. Arch. Lett..

[21] Li C., Song S.L., Dai H., Sidelnik A., Hari S.K.S., Zhou H. Locality-Driven Dynamic GPU Cache Bypassing. Proceedings of the 29th ACM on International Conference on Supercomputing.

[22] Chen X., Wu S., Chang L.-W., Huang W.-S., Pearson C., Wang Z., Hwu W.-M.W. Adaptive Cache Bypass and Insertion for Many-core Accelerators. Proceedings of the International Workshop on Engineering Simulations for Cyber-Physical Systems.

[23] Dai H., Li C., Zhou H., Gupta S., Kartsaklis C., Mantor M. A model-driven approach to warp/thread-block level GPU cache bypassing. Proceedings of the 53nd ACM/EDAC/IEEE Design Automation Conference (DAC).

[24] Gong X., Chen Z., Ziabari A.K., Ubal R., Kaeli D. TwinKernels: An execution model to improve GPU hardware scheduling at compile time. Proceedings of the IEEE/ACM International Symposium on Code Generation and Optimization (CGO).

[25] Pei Y., Yu L., Wu M., Chen T., Lou X., Zhang T. Two Methods for Combining Original Memory Access Coalescing and Equivalent Memory Access Coalescing on GPGPU. Proceedings of the 2016 13th International Conference on Embedded Software and Systems (ICESS).

[26] Kadam G., Zhang D., Jog A. BCoal: Bucketing-Based Memory Coalescing for Efficient and Secure GPUs. Proceedings of the 2020 IEEE International Symposium on High Performance Computer Architecture (HPCA).

[27] Rogers T.G., O’Connor M., Aamodt T.M. Divergence-aware warp scheduling. Proceedings of the 46th Annual IEEE/ACM International Symposium on Microarchitecture (MICRO-46).

[28] Sethia A., Jamshidi D.A., Mahlke S. Mascar: Speeding up GPU warps by reducing memory pitstops. Proceedings of the 2015 IEEE 21st International Symposium on High Performance Computer Architecture (HPCA).

[29] Do C.T., Choi H.J., Chung S.W., Kim C.H. (2019). A novel warp scheduling scheme considering long-latency operations for high-performance GPUs. J. Supercomput..

[30] Liang Y., Xie X., Wang Y., Sun G., Wang T. (2017). Optimizing Cache Bypassing and Warp Scheduling for GPUs. IEEE Trans. Comput. Des. Integr. Circuits Syst..

[31] Li D., Rhu M., Johnson D.R., O’Connor M., Erez M., Burger D., Fussell D.S., Redder S.W. Priority-based cache allocation in throughput processors. Proceedings of the 2015 IEEE 21st International Symposium on High Performance Computer Architecture (HPCA).

[32] Kim H., Hong S., Lee H., Seo E., Han H. Compiler-assisted GPU thread throttling for reduced cache contention. Proceedings of the 48th International Conference on Parallel Processing.

[33] Fang J., Wang M., Wei Z. (2020). A memory scheduling strategy for eliminating memory access interference in heterogeneous system. J. Supercomput..

[34] Koo G., Oh Y., Ro W.W., Annavaram M. Access Pattern-Aware Cache Management for Improving Data Utilization in GPU. Proceedings of the 2017 ACM/IEEE 44th Annual International Symposium on Computer Architecture (ISCA).

[35] Kloosterman J., Beaumont J., Wollman M., Sethia A., Dreslinski R., Mudge T., Mahlke S. WarpPool. Proceedings of the 48th International Symposium on Microarchitecture.

[36] Grauer-Gray S., Xu L., Searles R., Ayalasomayajula S., Cavazos J. (2012). Auto-tuning a high-level language targeted to GPU codes. Innov. Parallel Comput. (InPar).

[37] Che S., Boyer M., Meng J., Tarjan D., Sheaffer J.W., Lee S.-H., Skadron K. Rodinia: A benchmark suite for heterogeneous computing. Proceedings of the 2009 IEEE International Symposium on Workload Characterization (IISWC).

